# Diversity in the structures and ligand-binding sites of nematode fatty acid and retinol-binding proteins revealed by Na-FAR-1 from *Necator americanus*

**DOI:** 10.1042/BJ20150068

**Published:** 2015-10-16

**Authors:** M. Florencia Rey-Burusco, Marina Ibáñez-Shimabukuro, Mads Gabrielsen, Gisela R. Franchini, Andrew J. Roe, Kate Griffiths, Bin Zhan, Alan Cooper, Malcolm W. Kennedy, Betina Córsico, Brian O. Smith

**Affiliations:** * Instituto de Investigaciones Bioquímicas de La Plata, CONICET-UNLP, Facultad de Ciencias Médicas, calles 60 y 120, 1900-La Plata, Argentina; † Institute of Infection, Immunity and Inflammation, University of Glasgow, Glasgow G12 8QQ, U.K.; ‡ Institute of Biodiversity, Animal Health and Comparative Medicine, University of Glasgow, Glasgow G12 8QQ, U.K.; § National School of Tropical Medicine and Department of Pediatrics, Section of Tropical Medicine, Baylor College of Medicine, Houston, TX 20031, U.S.A.; ║ School of Chemistry, University of Glasgow, Glasgow G12 8QQ, U.K.; ¶ Institute of Molecular, Cell & Systems Biology, University of Glasgow, Glasgow G12 8QQ, U.K.

**Keywords:** fatty acid-binding protein, *Necator americanus*, nematode, nuclear magnetic resonance (NMR), parasite, protein structure, retinol-binding protein, X-ray

## Abstract

Fatty acid and retinol-binding proteins (FARs) comprise a family of unusual α-helix rich lipid-binding proteins found exclusively in nematodes. They are secreted into host tissues by parasites of plants, animals and humans. The structure of a FAR protein from the free-living nematode *Caenorhabditis elegans* is available, but this protein [*C. elegans* FAR-7 (Ce-FAR-7)] is from a subfamily of FARs that does not appear to be important at the host/parasite interface. We have therefore examined [*Necator americanus* FAR-1 (Na-FAR-1)] from the blood-feeding intestinal parasite of humans, *N. americanus*. The 3D structure of Na-FAR-1 in its ligand-free and ligand-bound forms, determined by NMR (nuclear magnetic resonance) spectroscopy and X-ray crystallography respectively, reveals an α-helical fold similar to Ce-FAR-7, but Na-FAR-1 possesses a larger and more complex internal ligand-binding cavity and an additional C-terminal α-helix. Titration of *apo*-Na-FAR-1 with oleic acid, analysed by NMR chemical shift perturbation, reveals that at least four distinct protein–ligand complexes can be formed. Na-FAR-1 and possibly other FARs may have a wider repertoire for hydrophobic ligand binding, as confirmed in the present study by our finding that a range of neutral and polar lipids co-purify with the bacterially expressed recombinant protein. Finally, we show by immunohistochemistry that Na-FAR-1 is present in adult worms with a tissue distribution indicative of possible roles in nutrient acquisition by the parasite and in reproduction in the male.

## Abstract (precis)

*Necator americanus* fatty acid and retinol-binding protein-1 (Na-FAR-1) is an abundantly expressed FAR from a parasitic hookworm. The present work describes its tissue distribution, structure and ligand-binding characteristics and shows that Na-FAR-1 expands to transport multiple FA molecules in its internal cavity.

## INTRODUCTION

Lipids such as acids (FAs) and retinoids are relatively insoluble in water, can be susceptible to oxidation and are potentially damaging to membranes in their free form. Consequently, they are usually transported within proteins or protein-mediated lipid aggregates. Examples of vertebrate FA and retinoid transporter proteins include serum albumins (∼64 kDa), which bind a range of compounds, lipocalins (∼20 kDa), which are found in many secretions and the proteins of the cytoplasmic FA-binding/cellular retinol-binding/cellular retinoic acid-binding protein (FABP/CRBP/CRABP) family (∼14 kDa) that are confined to the cytoplasm, where they may bind FAs, retinol or retinoic acid, depending on the isoform. These proteins are variously involved in transport and storage of lipids and also in the delivery of small signalling lipids to their destinations [1].

Nematodes, both free-living and parasitic to plants and animals, exhibit several additional types of lipid-binding proteins that are not found in other phyla. Prominent examples include the nematode polyprotein antigens (NPAs), such as ABA-1, whose structure has previously been solved [2]. These are synthesized as large polypeptide precursors that are post-translationally processed down to multiple copies of small lipid-binding proteins of ∼14 kDa [3–7]. Other examples include the nemFABPs (nematode FABPs), which are similar to the intracellular FABP/CRBP/CRABP family of proteins, but in nematodes have structural modifications found in no other group of animals and are not confined to the cytosol [8,9]. The subject of the present paper is an unusual class of lipid-binding protein, the FA and retinol-binding proteins (FARs). These occur in several isoforms of ∼20 kDa, eight having been found encoded within the genome of the free-living species *Caenorhabditis elegans*, each of which binds FAs and retinol to varying extents [10]. There are as yet uncharted numbers of FAR isoforms in parasitic nematodes, but FARs have drawn attention because they are secreted by both plant- and animal-parasitic species and their encoding transcripts are relatively abundant [11–18]. They have also proven useful for serodiagnosis, have shown promise in experimental vaccines [19,20] and have been proposed to facilitate infection by manipulating host lipid-mediated defences [21,22]. At least one FAR has been shown to bind an anthelmintic drug, these drugs being typically hydrophobic and so may require carrier proteins to conduct them to their site of action within parasites [23]. The definitive role of FARs in parasitism is not known, but their presence in secretions of the worms, coupled with their ligand-binding propensities, suggests roles for them either in acquiring lipids from the hosts, or in delivering or in sequestering signalling lipids and so modifying the tissues the parasites occupy or the immune responses against them.

The structure of one FAR protein, *C. elegans* FAR-7 (Ce-FAR-7), has been solved by X-ray crystallography, revealing a helix-rich structure that is unlike any type of lipid-binding protein previously described [24]. The mRNA for Ce-FAR-7 does not encode a secretory signal peptide and its amino acid sequence indicates that it is in a different subfamily of FARs from those that are secreted from the synthesizing cell. We set out to confirm the expression pattern of a secreted FAR protein from a parasite and characterize its structure and ligand-binding characteristics. The protein Na-FAR-1 derives from the blood feeding intestinal hookworm of humans, *N. americanus*. This parasite and the other hookworm of humans, *Ancylostoma duodenale*, together infect over 300 million people worldwide [25,26], causing considerable morbidity, together with adverse social and economic consequences. We have determined the structure of Na-FAR-1 by both X-ray crystallography and NMR spectroscopy in solution. We find that it has a similar overall fold to Ce-FAR-7, indicating that FARs are structurally conserved despite considerable sequence diversity. But, in addition to the structural differences between Ce-FAR-7 and Na-FAR-1, we find that their ligand-binding sites differ significantly in position and form.

## EXPERIMENTAL

### Protein expression and purification

Recombinant Na-FAR-1 (rNa-FAR-1) was expressed in BL21 (λDE3) *Escherichia coli* cells as described [27]. For native crystallographic studies, Na-FAR-1 was purified to homogeneity, as previously described [28], from cells grown in LB media. Selenomethionine-labelled protein was purified from B834 cells grown in M9 minimal medium supplemented with a cocktail of free amino acids (each 0.5 g·l^−1^) and selenomethionine (50 mg·l^−1^; Generon).

For NMR studies, samples of unlabelled, ^15^N-labelled and ^13^C^15^N-labelled protein were purified by nickel-affinity, size exclusion and reverse-phase chromatographies, as described [27], from cells grown in M9 minimal medium containing ^15^NH_4_Cl, [^13^C_6_]-glucose or their unlabelled equivalents.

### Western blotting and immunolocalization of Na-FAR-1

Antiserum prepared against recombinant Na-FAR-1 was raised in three rabbits by subcutaneous injection with 0.7 mg of purified recombinant Na-FAR-1 in Freund's complete adjuvant. Antiserum was tested by ELISA and Western blot analysis against the recombinant protein. To analyse the expression of Na-FAR-1 in the worm, soluble extracts of adult *N. americanus*, as well as recombinant proteins (each 100 ng) of homologues from *Ancylostoma caninum* FAR-1 (Ac-FAR-1); *Brugia malayi* FARs (Bm-FAR-1 and Bm-FAR-2) and the unrelated protein, recombinant Ac-SPI (serine protease inhibitor from *A. caninum*) were separated on a 4%–20% gradient SDS precast polyacrylamide gel (Invitrogen) and subsequently electrotransferred on to a PVDF membrane (Millipore). Rabbit anti-Na-FAR-1 serum was diluted 1:5000 into PBS (phosphate buffered saline), pH 7.4, with 0.05% Tween-20 and incubated with transferred membrane for 2 h. Horseradish peroxidase-conjugated goat anti rabbit-IgG was used as secondary antibody and ECL was used to develop the reaction (GE).

To determine the tissue-specific localization of Na-FAR-1, adult *N. americanus* worms were prepared as previously described [29]. Briefly, adult worms were collected from the intestines of hamsters infected with *N. americanus* L3 [third (infective) larval stage of a nematode] for 45 days and fixed with 10% formalin. The fixed worms were sectioned and mounted on glass slides. The non-specific binding sites on worm sections were blocked with 5% FBS in PBS for 1 h. The rabbit anti-Na-FAR-1 serum was applied (1:500 dilution) to each tissue section and incubated for 2 h at room temperature in a humidified chamber. Pre-immune rabbit serum at the same dilution was used as a negative control. Sections were washed six times for 5 min each in PBS and probed with anti-rabbit Cy3-conjugated IgG (Rockland). Sections were viewed under a Nikon TE-2000 Inverted fluorescence microscope using a 550 nm excitation filter block and emission at 565 nm.

### Crystallization, data collection, processing and structure solution

We have shown previously that Na-FAR-1 crystallizes in two crystal forms, one of which (form 2) shows significant twinning [28]. Here, in order to obtain phasing information, selenomethionine-substituted protein was purified and crystallized, selecting only the cubic crystal form 1. Crystals were frozen in a stream of cool nitrogen gas (100 K) and brought to the Diamond Light Source, station I04 (DLS) for X-ray diffraction data collection.

Data were collected at 0.7° increments per image, for a total of 200 images [wavelength 0.9793 Å (1 Å=0.1 nm)] and processed by the automatic processing routines fast_dp, which utilized XDS [30], POINTLESS and SCALA [31]. The structure was solved using the SAS protocol of Auto-Rickshaw [32]. The input diffraction data were prepared and converted for use in Auto-Rickshaw, using programs of the CCP4 suite [33]. F_A_ values were calculated using the program SHELXC [34]. Based on an initial analysis of the data, the maximum resolution for sub-structure determination and initial phase calculation was set to 2.14 Å based on the scaling statistics and the increase in *R*_meas_ in the highest resolution bin. All the four heavy atoms requested were found using the program SHELXD [35]. The correct handedness for the substructure was determined using the programs ABS [36] and SHELXE [34]. Initial phases were calculated after density modification using the program SHELXE. 83.9% of the model was built using the program ARP/wARP [37]. Despite a solvent content above 70%, only one copy of the protein was observed in the asymmetric unit. The model was completed by hand using COOT [38] and iterative rounds of BUSTER [39]. Ligands and water were added using COOT. Based on its prevalence in the lipids co-purifying with recombinant Na-FAR-1, palmitate was fitted into unoccupied electron density within the binding pocket. Further electron density that may represent additional ligand molecules was observed, but as the density was incomplete and not at full occupancy, it was left unmodelled. The geometry of the finished models was validated using Molprobity [40].

### Preparation of NMR samples

*Apo*-Na-FAR-1 samples were delipidated by reverse phase-HPLC (high performance liquid chromatography) as previously described [27] using a C_8_-silica stationary phase and a water/acetonitrile gradient in the presence of 0.1% trifluoroacetic acid as the mobile phase. Samples were lyophilized, reconstituted and concentrated to approximately 0.6 mM in 20 mM sodium phosphate, pH 7.20. ^2^H_2_O was added to a final concentration of 5% (v/v). The sample used for residual dipolar coupling (RDC) measurements was prepared by partial alignment of the uniformly ^13^C^15^N-enriched Na-FAR-1 in a solution of magnetically aligned filamentous Pf1 bacteriophage [41] (ASLA biotech). The degree of alignment was evaluated by measuring the ^2^H quadrapolar splitting in the HDO resonance. After testing several conditions, a NaCl concentration of 300 mM with 9 mg·ml^−1^ of bacteriophage and 300 μM protein was selected.

### NMR spectroscopy and analysis

All spectra were recorded at 311 K on a Bruker AVANCE 600 MHz spectrometer equipped with TCI cryoprobe. Resonances were assigned, as described previously [27]. All spectra were processed in AZARA (Wayne Boucher, http://www.bio.cam.ac.uk/azara). Maximum entropy reconstruction [42] was used to enhance resolution of the indirect dimensions of 3D experiments. Spectra were analysed with CCPNmr analysis software [43]. Frequency-based methods were employed to measure ^1^D_NH_ [44] and ^1^D_CαHα_ [45] couplings from in-phase/antiphase ^15^N-HSQC (heteronuclear single-quantum correlation spectroscopy) spectra. Distance restraints for structure calculations were derived from 3D ^15^N-NOESY-HSQC [46] and 3D ^13^C-NOESY-HSQC spectra, each recorded with 100 ms mixing time.

### Structure calculations from NMR-based restraints

Distance restraints were derived from NOESY (nuclear Overhauser effect spectroscopy) cross-peaks with the initial mapping from normalized intensity to distance and grouped in distance bins. NOE distance restraints were incorporated in restrained MD calculations using the ambiguous distance restraints formalism [47] using ARIA 2.3 [48] and CNS [49]. Loose backbone dihedral restraints for regions of regular secondary structure predicted based on secondary chemical shifts by DANGLE [50] were incorporated during the high temperature phases of the simulations but omitted during the final cooling phase. RDC and hydrogen bond restraints were then introduced. The average RDC alignment tensor was estimated from the ensemble calculated using only NOEs with PALES [51] and used to incorporate the RDC restraints via the SANI potential [52] in square-well mode. The 20 structures that best satisfy the experimental restraints were chosen from 100 structures generated in the final iteration and refined in explicit water [53]. The quality of these structures was analysed using PROCHECK_nmr [54] and their co-ordinates deposited in the Protein Data Bank under accession code 4UET. Structure figures were generated using PyMOL (http://www.PyMOL.org).

### NMR relaxation measurements

^15^N-relaxation time constants, *T*_1_ and *T*_2_ were assessed using the method of Kay [55–57] at a field strength of 14.1 T. Relaxation delays for assessment of *T*_1_ were 101, 601, 1001 and 1401 ms whereas those for *T*_2_ were 17, 34, 68, 102 and 136 ms. Selected time points in each series were repeated in order to estimate the inherent error in calculation of cross-peak intensities. Relaxation times *T*_1_ and *T*_2_ were calculated using non-linear least squares fitting. Collection of ^15^N-HSQC-heteronuclear NOE experiments with and without saturation allowed extraction of ^1^H,^15^N NOE values. Both saturation and reference experiments were repeated for the purpose of error estimation.

### Ligand-binding analysis by chemical shift perturbation

In order to examine Na-FAR-1’s ligand binding properties by NMR, the chemical shift changes induced in ^15^N HSQC spectra of the protein by the presence of increasing amounts of ligand were analysed. Double-labelled ^13^C-^15^N recombinant Na-FAR-1 (0.4 mM) was titrated by sequential addition of small volumes of unlabelled sodium oleate (Sigma–Aldrich) from a 125 mM stock solution in water (pH ∼9). Triple resonance experiments were recorded at selected points of the titration in order to assign the displaced cross-peaks. The titration started by addition of 0.5 molar equivalent of the ligand and was continued until turbidity due to the presence of insoluble oleic acid was observed.

### Lipid extraction and analysis

Total lipids were extracted according to the methodology described by [58] and [59], with minor modifications. For unstripped Na-FAR-1 (no RP-HPLC purification), lipids were extracted from 15 mg of protein and compared with control *apo*-Na-FAR-1 (RP-HPLC purified protein). For comparison, approximately 3 ml of culture of *E. coli* BL21 (λDE3) cells were lysed by sonication. Each sample was mixed with 15 ml of CHCl_3_–CH_3_OH (2:1) and vigorously shaken for 15 min in an ice bath. The homogenate was washed with 250 μl of 2.9% (w/v) NaCl solution. After agitation, the phases were separated by centrifugation and the upper, aqueous phase discarded. The lower phase containing lipids was recovered and dried under a stream of N_2_ gas, re-dissolved in CHCl_3_ and stored at–20°C under N_2_ gas until analysis.

Lipid classes bound to Na-FAR-1were analysed by TLC (thin layer chromatography) on silica gel plates Si250 (J.T.Baker) with the methodology and solvent systems described by [59]. Lipid samples obtained from *holo*-Na-FAR-1 and *E. Coli* lysates controls and standards were spotted on TLC plates (20×20 cm) previously activated at 100°C for 30 min and developed with methyl acetate–isopropanol–chloroform–methanol–0.25% KCl (25:25:25:10:9, by volume) for polar lipids and hexane/diethyl-ether/acetic acid (80:20:1, by volume) for neutral lipids.

Non-esterified FA and phospholipid (PL) TLC spots were scraped and extracted from the silica with pure chloroform for FAs and chloroform–methanol–water for PLs respectively. FA composition was analysed by GC (gas chromatography)–MS of their methyl ester derivatives, prepared with BF_3_-methanol according to the method of Morrison and Smith [60] as described previously [61]. The individual FA methyl ester peaks were identified by comparison of their retention times with those of standards and by their mass spectra.

### Fluorescence spectroscopy

Fluorescence experiments were performed with a Fluorolog-3 Spectrofluorometer (Horiba-Jobin Yvon). Buffer alone was used to correct for Raman and background scattering. RP-HPLC delipidated Na-FAR-1 was employed in all ligand-binding experiments.

The ligand-binding capacity of Na-FAR-1 was investigated with the fluorescent ligands 11-(dansylamino)undecanoic acid (DAUDA) and retinol. Stock solutions (10 mM) were prepared in ethanol and then diluted in PBS for use in the assays. Retinol solutions were diluted in ethanol and added directly to the cuvette to minimize degradation.

The FA chain length preference of rNa-FAR-1 was tested by displacement of the fluorescent ligand DAUDA as described [13]. Binding of non-fluorescent ligands was detected by a reversal of the wavelength shift and a decrease in fluorescence emission intensity on equal additions of test ligands to a DAUDA–rNa-FAR-1 complex recorded at the peak fluorescence emission wavelength of DAUDA in the protein (470 nm). The concentration of Na-FAR-1 in the cuvette was 1.5 μM. DAUDA ethanol stock solution was diluted 1:10000 in PBS for use in the assays at 1 μM. Stock solutions of all the non-fluorescent competitors were made to approximately 10 mM in ethanol, then diluted in PBS for use in the assays.

## RESULTS AND DISCUSSION

### Na-FAR-1

**Figure 1:**
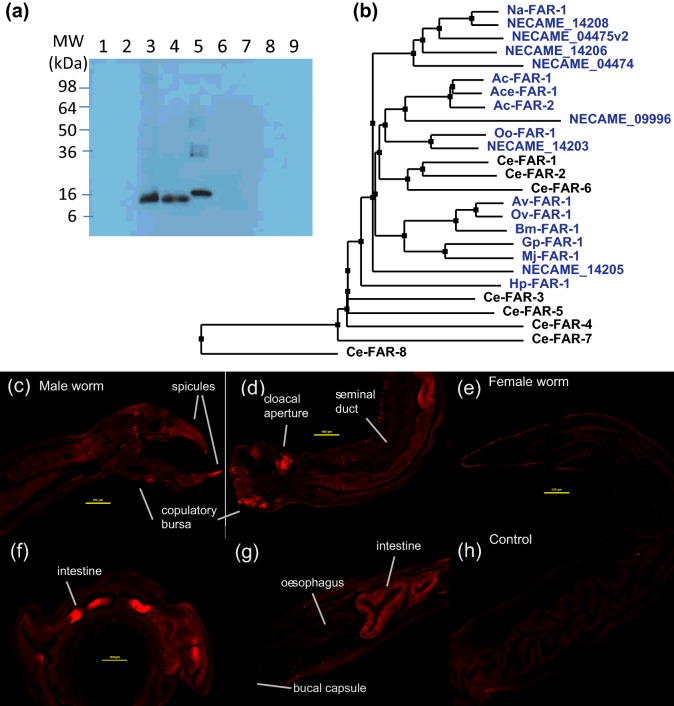
FAR protein relationships and Na-FAR-1 expression (**a**) Western blot with anti-Na-FAR-1 serum that specifically recognizes native Na-FAR-1 in *N. americanus* extracts (lane 3, 0.5 μg) and excreted/secreted (ES) products (lane 4, 0.5 μg) at an approximate *M*_r_ of 14 kDa, but not in L3 extracts (lane 1) and L3 ES products (lane 2) at the same loading, indicating the specific expression of Na-FAR-1 in adult stage as a secreted protein. The antiserum also recognized the recombinant Na-FAR-1 at 16 kDa (with His-tag, lane 5, 20 ng). There was no cross-reaction with FAR homologues from dog hookworm *A. caninum* (Ac-FAR-1, lane 6); *B. malayi* (Bm-FAR-1/lane 7, Bm-FAR-2/lane 8) and non-relevant recombinant protein Ac-SPI (lane 9) loaded at the same amount (20 ng). (**b**). Neighbour joining tree of Na-FAR-1 and other nematode FAR proteins. The tree was generated in *jalview 2.8* [69] using the BLOSUM 62 matrix from a *T-coffee WS* sequence alignment [70] with FAR protein amino acid sequences of: *N. americanu*s recently identified FAR proteins (NECAME_09996, NECAME_04475, NECAME_04474, NECAME_14206, NECAME_14205 and NECAME_14203), the free living nematode *C. elegans* (Ce-FAR-1 to Ce-FAR-8), the human parasitic nematodes *O. volvulus* (Ov-FAR-1), *B. malayi* (Bm-FAR-1), animal parasitic nematodes *A. caninum* (Ac-FAR-1 and Ac-FAR-2), *A. ceylanicum* (Ace-FAR-1), *O. ostertagi* (Oo-FAR-1) and *H. polygyrus* (Hp-FAR-1) and the plant parasitic nematodes *G. pallida* (Gp-FAR-1) and *M. javanica* (Mj-FAR-1). All the parasite proteins are coloured blue. See Supplementary Figure S1 for the multiple sequence alignment from which the tree was constructed. (**c–g**) Localization of Na-FAR-1 within adult male (**c** and **d**) and female (**e**–**g**) worms. Indirect immunofluorescence localization with rabbit anti-Na-FAR-1 serum stains the intestinal cells of adult *N. americanus* worms. Na-FAR-1 was also detected on the copulatory bursa and cloacal aperture of male worms. (**h**) Control carried out using pre-immune serum. Scale bars represent 100 μm.

Na-FAR-1 had been identified in a gene survey of the *N. americanus* transcriptome under the name *N. americanus* LBP-20 (sequence ID NAC00128) [62]. LBP-20 proteins are currently named FAR due to their capacity for binding FAs and retinol and Na-FAR-1 was renamed accordingly [63]. More recently, the genome of *N. americanus* has been published and at least six FAR proteins have been recognized (Figure 1; Supplementary Figure S1), including Na-FAR-1, under the sequence name NECAME_14208 [64]. Na-FAR-1 cDNA predicts a 19364.57 Da protein with a 14 amino acid secretion signal peptide predicted by SignalP [65]. The post-translational removal of the leader peptide would yield a mature protein of 155 amino acids with a molecular mass of 17082.49 Da.

A phylogenetic tree was constructed to show the relationship between Na-FAR-1 and FARs from other species (Figure 1b). The tree was constructed with amino acid sequences omitting any leader peptides identified by SignalP. The sequences included those of *C. elegans* (Ce-FAR-1–8), the human parasitic nematodes *Onchocerca volvulus* FAR-1 (Ov-FAR-1), *B. malayi* (Bm-FAR-1), animal parasitic nematodes *A. caninum* (Ac-FAR-1 and Ac-FAR-2), *Ancylostoma ceylanicum* FAR-1 (Ace-FAR-1), *Ostertagia ostertagi* FAR-1 (Oo-FAR-1) and *Heligmosomoides polygyrus* FAR-1 (Hp-FAR-1) and the plant parasitic nematodes *Globodera pallida* FAR-1 (Gp-FAR-1) and *Meloidogyne javanica* FAR-1 (Mj-FAR-1). FAR transcript levels in some of these parasites are notably high, particularly in the parasitic stages of life cycles [11–18,62,66]. Figure 1 shows that most FAR proteins known from parasites group together, including those from *N. americanus*, and a subset of FARs from *C elegans* (Ce-FAR-1, 2 and 6). As noted above, Ce-FAR-7, the only FAR protein whose structure was previously known, falls in a subfamily of FARs distant from all of the FAR proteins from parasites [10] (Figure 1b).

### Na-FAR-1 localization within the parasite

Na-FAR-1-encoding mRNA had been detected in adult and larval L4 *N. americanus* life stages [62] and analysis of differences in gene expression between infective larvae (iL3) and adult parasitic stages shows that Na-FAR-1 is mainly expressed in the adult, blood-feeding stage [64]. Immunohistochemistry revealed that Na-FAR-1 protein occurs in the cloacal aperture and copulatory bursa of male worms (Figures 1c and 1d) and in the intestine (Figures 1f and 1g). The presence of Na-FAR-1 in the parasite's intestinal cells may indicate involvement in transportation and storage of lipids derived from host blood and its location in copulatory bursa of male worms suggests its function may additionally be related to reproduction. Na-FAR-1 was detected in the intestinal cells but not the reproductive structures of females (Figure 1e).

### 3D structure of Na-FAR-1

The 3D structure of Na-FAR-1 was determined by both protein X-ray crystallography and solution state NMR spectroscopy. Na-FAR-1, which had not been subjected to reverse phase HPLC purification to strip out co-purifying ligands, crystallized as previously reported and diffracted to 2.14 Å [28]. Molecular replacement using the Ce-FAR-7 structure proved unsuccessful and we therefore obtained the crystal structure of Na-FAR-1 in complex with co-purifying ligands, hereafter referred to as *holo*-Na-FAR-1, by anomalous dispersion from crystals of Se-Met-labelled protein (Table 1). In contrast, NMR spectra of unstripped Na-FAR-1 in solution, like those of other FAR proteins we have tested, were characterized by broad signal peaks indicative of multiple conformations and/or conformational exchange. However, stripped Na-FAR-1 gave good solution NMR spectra and the structure of *apo*-Na-FAR-1 was determined from a total of 7289 NOE-derived distance restraints, 316 dihedral angle restraints and restraints derived from 177 RDCs (101 ^1^D_NH_ and 76 ^1^D_CaHa_) observed in a sample that had been partially aligned in Pf1 filamentous bacteriophage (Table 2).

**Table 1 Data:** collection, phasing and structure refinement statistics for *holo*-Na-FAR-1 determined by X-ray crystallography (PDB:4XCP) Numbers in brackets indicate values in the highest resolution bin (2.27–2.14 Å). Abbreviation: PDB, protein structure database.

Data collection	
Space group	P432
Unit cell	*a*=*b*=*c*=121.123
Wavelength (Å)	0.9793
Resolution (Å)	29.38-2.14
Observed reflections	502169 (17556)
Unique reflections	17353 (1212)
Completeness	99.91 (97.0)
Multiplicity	28.9 (14.5)
*R*_meas_ (%)	14.2 (78.5)
*R*_pim_ (%)	2.6 (20.3)
*R*_anom_ (%)	5.2 (17.5)
<I/σ(I)>	25.5 (4.2)
Wilson B (Å)^2^	30.11
Number of Selenomethionines found	4

Refinement	

*R*_work_ (%)	20.5
*R*_free_ (%)	22.4
Protein residues/atoms	155 (1213)
Water molecules	139
Palmitic acid	1
RMSD bond lenghts (Å)/angles (°)	0.01/0.99
Average isotropic thermal parameters (Å^2^)	
Main chain/side chain atoms	25.06/33.69
Water molecules	38.09
Palmitic acid	38.11
Ramachandran analysis	
% Favoured regions	98.05
% Outliers	0.65

**Table 2:** Structural statistics for the ensemble of 20 *apo*-Na-FAR-1 structures determined by NMR spectroscopy

NOE distance restraints	
Total NOE	7097
Ambiguous	3443
Unambiguous	3654
Intra-residue	1548
Inter-residue	2106
Sequential (|i−j|=1)	833
Short range (1 < |i−j| ≤ 4)	756
Long range (|i−j| ≥ 5)	517
Violations > 0.5 Å	0.3
Violations > 0.3 Å	1.6
Distance restraints RMSD (Å)	0.016

Coordinate RMSD (Å)	

Backbone heavy atoms	0.597
All heavy atoms	1.012

Parameter RMSD from idealized geometry (mean ± S.D.)	

Bond lengths (Å)	0.00326±1.4×10^−4^
Bond angles (°)	0.595±0.018
Impropers (°)	1.57±0.07

RDC restraints	

^1^D_NH_	102
^1^D_CαHα_	76
RDC Q factor	0.164
Dihedral angles	312

Ramachandran statistics (%)	

Most favoured	92.9
Additionally allowed	6.0
Generously allowed	0.8
Disallowed	0.3

**Figure 2:**
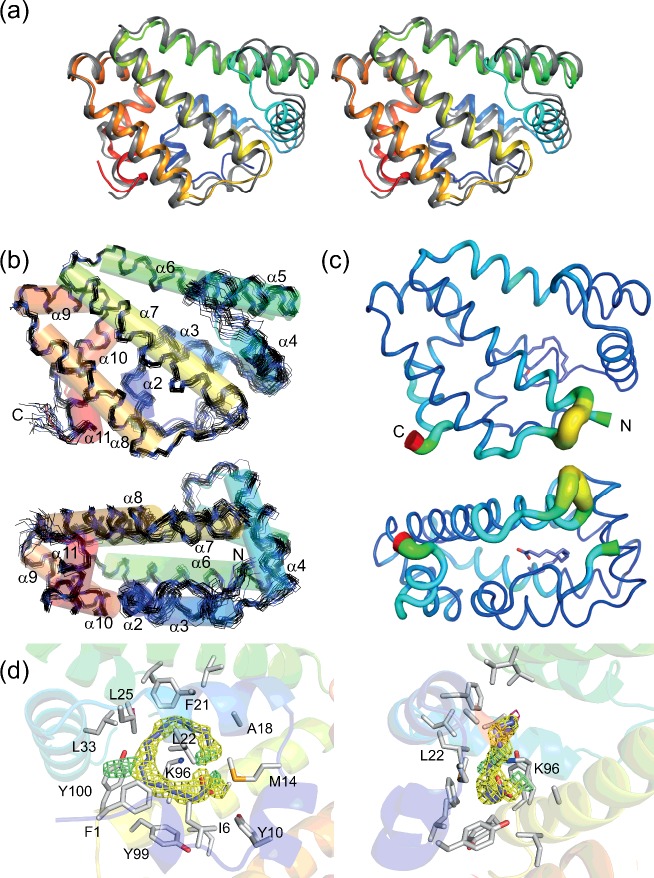
The structure of Na-FAR-1 (**a**) Stereoscopic view (wall-eyed) of the superimposed *apo-* and *holo*-Na-FAR-1 structures shown in cartoon form. The *apo* structure determined by NMR is coloured from blue at the N-terminus through to red at the C-terminus. The *holo* form determined by X-ray crystallography is shown in grey. (**b**) The ensemble of the 20 lowest energy structures of *apo*-Na-FAR-1 shown in two orientations related by a 90° rotation. (**c**) The *holo*-Na-FAR-1 structure shown in b-factor putty representation with the palmitate shown as sticks. (**d**) Expanded view of the bound palmitate and its environment with 2Fo-Fc electron density within 4 Å of the ligand displayed as a mesh at 1σ and shaded yellow and Fo-Fc difference density displayed at ±3σ (green and red).

**Figure 3:**
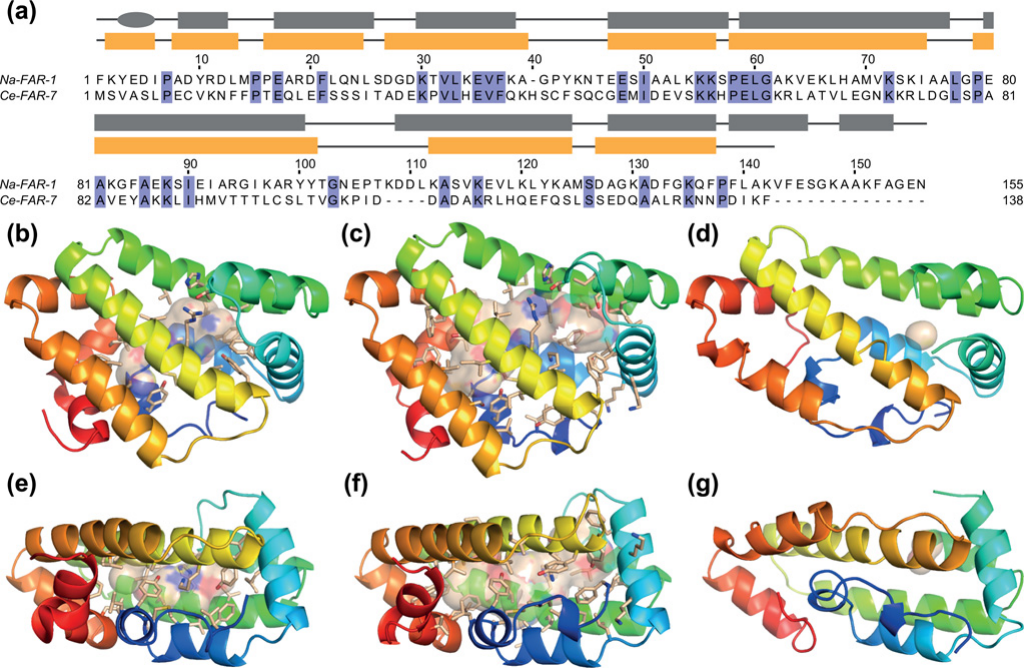
Na-FAR-1 cavities and comparison with Ce-FAR-7 (**a**) Sequence alignment of Na-FAR-1 and Ce-FAR-7. Secondary structure elements are indicated in boxes (α-helix) and lines (loops) coloured grey for Na-FAR-1 and yellow Ce-FAR-7. Comparison of Na-FAR-1 *apo* (**b** and **e**) and *holo* (**c** and **f**) forms with Ce-FAR-7 (**d** and **g**) shown in cartoon representation and coloured from blue (N-terminus) to red (C-terminus) viewed from two orientations related by a 90° rotation about the horizontal axis. Internal cavities accessible to a probe of 1.925 Å, equivalent to a methylene group are shown as transparent surfaces with the surrounding sidechains shown as sticks. (PDB accession codes Na-FAR-1 *apo* 4UET and *holo* 4XCP; Ce-FAR-7, 29WY)

The overall folds of the Na-FAR-1 structures in complex with ligands (*holo*) and without ligand (*apo*) are similar (Figure 2a–c). Superposition of the *holo* and *apo* structures gives a 1.814 Å co-ordinate RMSD (root-mean-square deviation) for all heavy atoms (1.579 Å for main chain heavy atoms only). Na-FAR-1 presents a wedge-shaped structure with two larger faces each of approximately 40 by 30 Å in area and of ∼17 Å in width at the wide end of the wedge. The fold is organized into 11 helices of various lengths, defining an internal cavity (Figure 3). The N-terminal helices, 3_10_ (residues 3–5), α2 (8–11) and α3 (16–24) are co-planar with the C-terminal helices α9 (126–135), α10 (137–144) and α11 (147–152) and together with one of the longest helices, α6 (57–75), form one large face of the wedge. The other two long helices, α7 (79–99) and α8 (107–122), are co-planar with α5 (45–55) and form the other large face. These two faces enclose an internal cavity that is closed at the thick end of the wedge by α4 (29–37), which is almost perpendicular to the long axis of the molecule. Hydrophobic residues are located almost exclusively on the inward-facing sides of the helices with their side chains pointing towards the internal cavity. Most of the polar and charged residues are on the external surface of the protein generating a predominately hydrophilic surface. The notable exceptions are Ser^88^, Lys^96^ and Tyr^100^ whose side chains are located within the cavity, whereas His^67^ and Arg^93^ stand on either sides of the largest opening to the cavity.

Despite their similarities, the structures of the *apo* and *holo* forms of Na-FAR-1 reveal significant differences that reflect structural alterations upon ligand binding. The internal cavity is much larger for the *holo* protein, achieved by a global outward displacement of the surrounding helices by a little over 1 Å compared with the *apo* form, with the biggest changes seen at the C-terminal end of helix α7 (Figure 2a). For the *apo* structure, the central cavity has a volume of approximately 1220 Å^3^ accessible to a 1.4 Å radius probe (equivalent to a water molecule) or 940 Å^3^ accessible to a 1.925 Å probe (equivalent to a CH_2_ group). In the *holo* form, the cavity is more than twice as big, reaching 2570 and 2170 Å^3^ for probes of 1.4 and 1.925 Å respectively.

The main part of the *apo*-Na-FAR-1 cavity is lined by the side chains of residues Leu^13^, Met^14^, Pro^15^, Ala^18^, Phe^21^, Leu^22^, Leu^33^, Phe^37^, Thr^45^, Val^63^, Leu^66^, His^67^, Val^70^, Ile^89^, Ala^92^, Arg^93^, Lys^96^, Tyr^100^ and Leu^139^, most of which are apolar, with polar groups contributed by the side chain of Tyr^100^ and backbone carbonyl of Leu^13^ and the positively charged Lys^96^. His^67^ and Arg^93^ are located at the entrance to the cavity. A further, less accessible volume is located beyond a pinch point between Ala^92^ and Leu^139^ and is lined by the predominately hydrophobic side chains of Tyr^10^, Ser^88^, Val^114^, Val^117^ and Phe^143^. The cavity has a single opening located on one side of the wedge between α6 and α7 and the α4–α5 loop that is surrounded by charged residues. In the *holo* structure the expanded cavity is lined by the same residues as in the *apo* structure, but with 21 additional, predominantly apolar, side chains contributing to the surface of the expanded cavity (Phe^1^, Ile^6^, Leu^25^, Lys^30^, Lys^34^, Val^36^, Ser^48^, Ile^49^, Leu^52^, Met^69^, Phe^84^, Ala^85^, Ile^91^, Ile^95^, Tyr^99^, Leu^110^, Ser^113^, Phe^132^, Phe^136^, Val^142^ and Ala^148^). In addition to the opening seen in the *apo* form, the cavity is also accessible to solvent via an opening between the α2–α3 and α9–α10 turns and α6. It is therefore possible that more than one region could be involved in ligand entrance or exit and the loop between α7 and α8, which exhibits a higher mobility than the average, could be a candidate region to participate in those processes. Within the cavity of the *holo* form, clear electron density was observed that is well fitted by a single FA molecule, containing 16 carbon atoms (Figure 2d). Since the predominant FA found in *E. coli* lipids is palmitate, we fitted this to the electron density. The palmitate appears to be curled into a C-shape around the side chain of Lys^96^ and contacts 11 other residues (Phe^1^, Ile^6^, Met^14^, Phe^21^, Leu^22^, Leu^25^, Leu^33^, Leu^66^, Ile^95^, Tyr^99^ and Tyr^100^). Additional electron density, possibly indicative of several other bound ligand molecules, is seen in the cavity of Na-FAR-1, but in light of the heterogeneous mixture of lipids associated with the protein and the poor quality of the density, we have not modelled them.

### Comparison of the structures of Na-FAR-1 and Ce-FAR-7

Ce-FAR-7 has previously been noted to lie in a distinct sub-group of FARs because it possesses two cysteines that may form a disulfide bond, has distinct lipid-binding characteristics, lacks a secretory leader peptide and because of the intron arrangement of its encoding gene [10,24]. The X-ray crystallographic structure of Ce-FAR-7 [24] displays similarities but several distinct differences to Na-FAR-1 that could relate to differences in their ligand binding and biological properties.

A structure-based sequence alignment (Figure 3a) reveals sequence identity between the two proteins at 32 positions (∼21%). Among these are: hydrophobic residues, Leu^77^ and Ala^81^ (Leu^78^ and Ala^82^ in Ce-FAR-7) that help to determine the angle between α6 and α7; Leu^33^, Leu^59^ (Leu^33^ and Leu^60^ in Ce-FAR-7) together with Leu^52^, which is replaced by another hydrophobic residue Val^53^, which are involved in maintaining the orientation between α6 and helices α4 and α5; a salt bridge between Glu^35^ and Lys^55^ (Glu^35^ and Lys^56^ in Ce-FAR-7) that helps to maintain the relative orientation of α4 and α5; and another salt bridge between Lys^73^ (Arg^74^ Ce-FAR-7) and Glu^17^ that helps to maintain the relative orientation of α6 and α3. Na-FAR-1 has an insertion of four residues (107–110) that constitute an additional turn of helix at the N-terminal end of α8. Conserved prolines Pro^7^, Pro^15^ and Pro^79^ initiate helices α2, α3 and α7, corresponding to Pro^7^, Pro^15^ and Pro^80^ in Ce-FAR-7, as they seem likely to, in other FARs. Ce-FAR-7 has an additional residue in the loop between helices α4 and α5 and it is 14 residues shorter at the C-terminal region, thereby lacking helices α10 and α11. In Ce-FAR-7 α1 comprises a full turn of α-helix, whereas Na-FAR-1 has a 3_10_ helix at this position. A comparison of the structures gives an RMSD of 1.711 Å (for a total of 636 atoms aligned) with the *apo* form and 1.646 Å (608 atoms aligned) with the *holo* form. The region comprising the α4–α5 joining loop, which might constitute part of the entrance to the cavity, was not modelled in the structure of Ce-FAR-7 due to insufficient electron density. It is likely that the lack of good definition is indicative of dynamic processes occurring in the α4–α5 loop, as we found by NMR for Na-FAR-1 (Supplementary Figure S2).

The estimated cavity for Ce-FAR-7 calculated with CASTp has a much smaller size than for both forms of Na-FAR-1, with a volume of 687.7 Å^3^ accessible to a probe equivalent to a water molecule and just to 390.0 Å^3^ for a probe equivalent to a CH_2_ group. In Ce-FAR-7, helices α2 and α3 approach α7 and α8 more closely, reducing the volume of the cavity and resulting in two distinct hydrophobic pockets. The majority of residues that line the Na-FAR-1 cavity and the Ce-FAR-7 P1 and P2 pockets are conserved or conservatively substituted. The exceptions are that the Na-FAR-1 cavity includes the side chains of more polar amino acids, such as Ser^88^, Arg^93^ and Lys^96^, which are found in positions occupied by Leu^89^, Thr^94^ and Leu^97^ respectively, in Ce-FAR-7.

### Ligand binding

**Figure 4:**
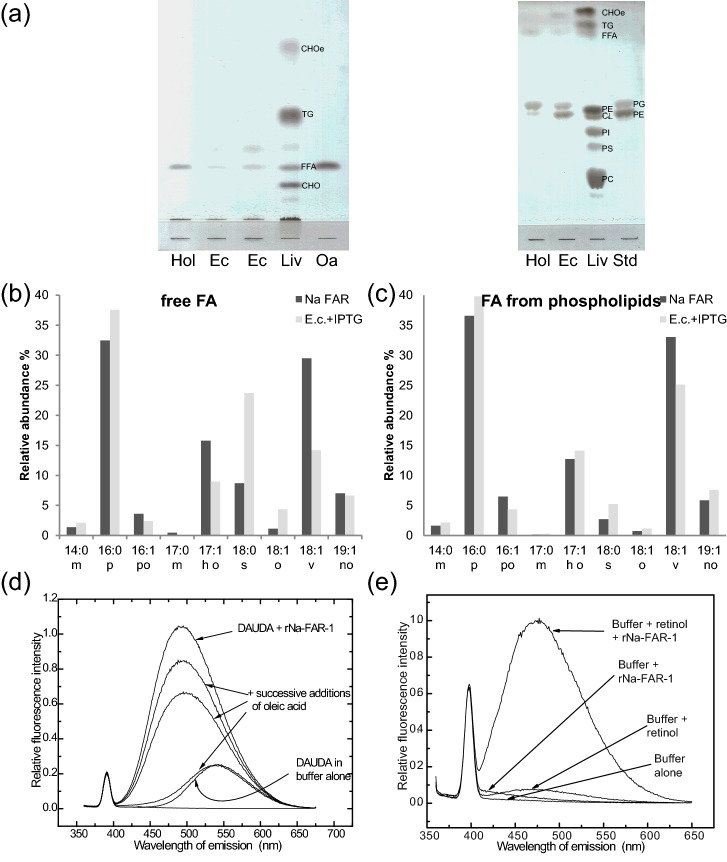
Lipid binding by recombinant Na-FAR-1 (**a**) Lipid fractions bound to bacterially-expressed Na-FAR-1 detected by TLC. Lipids were extracted from the protein and fractions were analysed by TLC in conditions for resolving separately neutral (left panel) and polar (right panel) lipid classes. Standards and samples applied to TLC plates were: Hol, Na-FAR-1 purified without an HPLC step; Ec, extract from whole *E. coli* cells; LIV, standard mix of lipids from rat liver homogenate; STD, *E. coli* whole extract. CHO, cholesterol; TG, triglycerides; CHOe, cholesterol esters; PC, phosphatidylcholine; PS, phosphatidylserine; PI, phosphatidylinositol; CL, cardiolipin. (**b**) GC–MS analysis of non-esterified FAs isolated from lipids associated with Na-FAR-1 purified from *E. coli* (dark grey) or found in *E. coli* extracts (light grey). FAs detected were: myristic (14:0), palmitic (16:0), palmitoleic (16:1), margarinic (17:0), heptadecenoic (17:1), stearic (18:0), oleic (18:1Δ9), vaccenic (18:1Δ11) and nonadecenoic acid (19:1). (**c**) FAs found in PLs isolated from purified Na-FAR-1 (dark grey) or found in *E. coli* extracts (light grey). (**d** and **e**) Fluorescent ligand binding by Na-FAR-1. (**d**) Fluorescence emission spectra of 1 μM DAUDA excited at 345 nm in buffer, on the addition of 1 μM Na-FAR-1 and after successive additions of oleic acid (81 nM, 810 nM and 8.1 μM). (**e**) Fluorescence emission spectra of 5 μl of 0.15 mM retinol in ethanol added to the fluorescence cuvette and excited at 350 nm and on the addition of 1.5 μM Na-FAR-1, compared with 1.5 μM protein in buffer and buffer alone.

In fluorescence-based ligand-binding assays, Na-FAR-1 bound the fluorescent FA analogue DAUDA and the naturally fluorescent lipid retinol (Figures 4d and 4e). The degree of blue shift in DAUDA fluorescence emission (from 543 nm in buffer to 480 nm) indicates Na-FAR-1 has a highly apolar binding site, as shown for other members of the FAR protein family [10]. It is also interesting to note that Ce-FAR-7 has shown no binding capacity for DAUDA [10] and binding to retinol showed no saturation [24]. The preference of Na-FAR-1 for FAs was investigated by the addition of FAs with different chain lengths to preformed protein–DAUDA complexes (Supplementary Figure S3), showing that DAUDA displacement was greatest with saturated FAs in the range C14:0–C19:0.

In order to determine the lipid classes bound to Na-FAR-1 in a cellular environment, a Folch extraction of the lipidic fraction of Na-FAR-1 purified from *E. coli* and subsequent TLC analyses were performed. The bacterial cytoplasm is not the natural environment of Na-FAR-1, but this approach could contribute to the understanding of its binding preferences. The lipid fraction bound to Na-FAR-1 exhibits heterogeneous lipid content (Figure 4a). Previous studies have shown that other members of the FARs bind retinol and FAs [10,13,15,19,21]. In addition, the present analysis reveals that the protein binds not only to FAs but also a broader range of lipid classes such as PLs.

The qualitative analysis conducted suggests the presence of polar lipids such as phosphatidylethanolamine (PE) and phosphatidylglycerol (PG). Esterification and analysis of the non-esterified FA and PL-derived FA methyl esters by GC–MS (Figures 4b and 4c) showed that in the lipids carried by Na-FAR-1, palmitate (16:0) was the most common FA. Examination of total *E. coli* lipids showed a similar FA composition.

**Figure 5:**
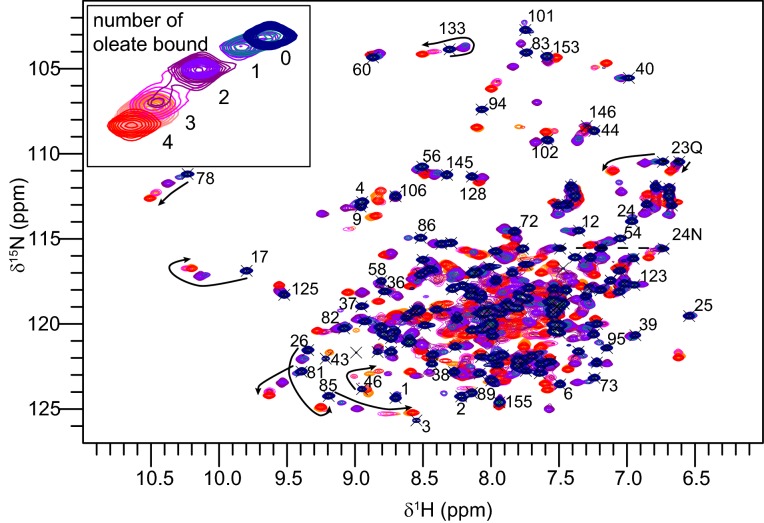
Titration of Na-FAR-1 with sodium oleate followed by NMR spectroscopy ^15^N HSQC spectra of 0.4 mM Na-FAR-1 with successive additions of sodium oleate. The spectrum of the *apo* protein is shown in dark blue, with spectra coloured through purple to red at the final protein–ligand ratio of 1:10. A subset of the backbone and side chain (lettered) assignments are shown and the trajectories of selected residues indicated with arrows. The inset shows an expanded view of the cross-peaks from Gly^78^ backbone amide with our interpretation of the protein–ligand stoichiometry responsible for each distinct cross-peak position.

Protein–ligand interactions were further investigated by titration of Na-FAR-1 with sodium oleate followed using NMR. The first step of the titration was made by adding of 0.5 molar equivalents of sodium oleate (Figure 5), at which point two cross-peaks originating from each shifted amide group were detected, one remaining at the original shift that corresponds to the *apo* protein conformation and another cross-peak at a different shift resulting from the protein conformation in the presence of one ligand bound. At subsequent stages in the titration, further chemical shift changes were observed, in each case resulting in the appearance of a new cross-peak and disappearance of the original. The ligand binding process therefore exhibited slow exchange behaviour through the addition of 1, 2 and 3 molar equivalents of oleate, which would suggest that the protein binds three ligands with high affinity. Beyond this, additional chemical shift changes were observed in the fast exchange regime from a 1:4 protein–ligand ratio where only one cross peak was seen for each amide group. The titration was carried on to a maximum protein–ligand ratio of 1:10 where it was limited by the precipitation of excess, unbound ligand. These results suggest that the protein binds up to at least four oleate molecules, three of them with high affinity in the sub micromolar range and a fourth with lower affinity. Substantial chemical shift changes were observed for residues throughout the protein, consistent both with ligand binding affecting the local environment of residues within the helices surrounding the cavity and with the changes in conformation of residues in the loops required to allow the structure to expand.

## CONCLUSION

The FAR proteins are unique to nematodes and we in the present study demonstrate the first structure of a member of this family from a parasitic species. FARs are present in the secretory products of parasites, which is apparent both for those that reside in the lumen of the gut and those that penetrate and reside in the host tissue of animals and plants [22,67]. This family of proteins comprises subfamilies that differ in their ligand-binding characteristics and in whether or not they are secreted from the cells that synthesize them. Transcripts encoding Na-FAR-1 are relatively abundant in the parasite from which it derives and the encoded polypeptide bears a secretory signal sequence. It falls into a new subfamily of the FARs that has not been found so far in any other group of nematodes, although the inventory of FARs is only comprehensive for those species for which genomic information is essentially complete and annotated (e.g. *C. elegans*).

It has been noted that all FAR protein sequences exhibit a consensus casein kinase II-type sequence that occurs in approximately the same place, despite the considerable degree of sequence diversity among FARs [19,24,67,68]. This argues that this site may have some significance in, for instance, altering the ligand-binding activity of the proteins or modifying interaction with other cellular factors such as cell surface receptors (host or parasite). There is currently no evidence that this site is phosphorylated naturally, though experiments with Ce-FAR-7 indicate that modification of this site does alter ligand binding. The previous study found that direct phosphorylation rendered the protein unstable in some unknown way, but when phosphorylation was mimicked by substitution of the presumed target threonine by an aspartic acid, the affinity of Ce-FAR-7 for retinol increased, although there was no change for FA. In both Ce-FAR-7 and Na-FAR-1, the amino acid side chain thought to be phosphorylated lies in a short unstructured region between two helices at one end of the molecule. In neither protein is the side chain of this residue oriented towards the internal cavity and nor in Ce-FAR-7 it is more distant from the internal surface than in Na-FAR-1. It is therefore not immediately clear how phosphorylation could affect the proteins’ ligand-binding characteristics unless structural changes result.

The differences in the size and shape of the internal cavities of Ce-FAR-7 and Na-FAR-1 are probably indicative of differences in their ligand selectivity. Both proteins bind retinol and FAs, but whether these compounds are relevant *in vivo* and whether other classes of ligand (such as PLs here found to be bound by Na-FAR-1) are important, remains to be established. The fact that FARs are commonly found in the secretions of parasitic nematodes bolsters confidence that they play an important role in parasitism. Parasites need to acquire nutrients from their hosts, but also need to defend themselves against immune defence reactions and some parasites induce modifications to the tissues they occupy. If FARs do bind signalling lipids, then it is conceivable that they are released by parasites in order to subvert or modify the hosts’ tissue and defence reactions. If so, then FARs would seem to be logical targets for vaccines, particularly against parasites that are embedded in host tissues. Development of drugs against them would need to take into account the possibility of interference with the hosts’ proteins and enzymes that transport or modify FAs and retinoids. But, it is entirely possible that we have yet to discover the true range of the ligand-binding propensities of FARs in nematodes. An understanding of their structures and how they vary between those secreted by different species of parasites could illuminate their roles in parasitism and suggest possible targets for therapeutic interventions.

## Abbreviations

Ace-FAR-1: *Ancylostoma ceylanicum* fatty acid- and retinol-binding protein-1
Ac-FAR-1: *Ancylostoma caninum* fatty acid- and retinol-binding protein-1
Ac-SPI: serine protease inhibitor from *Ancylostoma caninum*
Bm-FAR: *Brugia malayi* fatty acid- and retinol-binding protein
Ce-FAR-7: *Caenorhabditis elegans* fatty acid- and retinol-binding protein-7
CRABP: cellular retinoic acid-binding protein family
CRBP: cellular retinol-binding protein
DAG: diacylglycerol
DAUDA: 11-(dansylamino)undecanoic acid
FA: fatty acid
FABP: fatty acid-binding protein
FAR: fatty acid- and retinol-binding protein
GC: gas chromatography
Gp-FAR-1: *Globodera pallida* fatty acid- and retinol-binding protein-1
Hp-FAR-1: *Heligmosomoides polygyrus* fatty acid- and retinol-binding protein-1
HPLC: high performance liquid chromatography
HSQC: heteronuclear single-quantum correlation spectroscopy
L3: third (infective) larval stage of a nematode
Mj-FAR-1: *Meloidogyne javanica* fatty acid- and retinol-binding protein-1
Na-FAR-1: *Necator americanus* fatty acid- and retinol-binding protein-1
NMR: nuclear magnetic resonance
NOESY: nuclear Overhauser effect spectroscopy
NPA: nematode polyprotein antigen
Oo-FAR-1: *Ostertagia ostertagi* fatty acid- and retinol binding protein-1
Ov-FAR-1: *Onchocerca volvulus* fatty acid- and retinol-binding protein-1
PBS: phosphate buffered saline
PDB: protein structure database
PE: phosphatidylethanolamine
PG: phosphatidylglycerol
PL: phospholipid
RDC: residual dipolar coupling
RMSD: root-mean-square deviation
TLC: thin layer chromatography
